# Supplementary material for: PCBs in Chinstrap Penguins from Deception Island (South Shetland Islands, Antarctica)
Source: Toxics. 2025 May 24;13(6):430. doi: 10.3390/toxics13060430 (PMC12196938; doi:10.3390/toxics13060430)
Supplement: Supplementary file 1 [file toxics-13-00430-s001.zip › toxics-3602049-supplementary.pdf]

PCBs in Chinstrap penguins (Pygoscelis antarcticus) from Deception Island (South Shetland Islands, Antarctica)

Miguel Motas[a]; Silvia Jerez-Rodríguez[a]; José Manuel Veiga-del-Baño[b]; Juan José Ramos[c]; José Oliva[b]; Miguel Ángel Cámara[b]; Pedro Andreo-Martínez[b] *; Simonetta Corsolini[d]

[a] Department of Toxicology, Faculty of Veterinary, University of Murcia Regional Campus of International Excellence Campus Mare Nostrum, Campus of Espinardo, 30100 Murcia, Spain

[b] Department of Agricultural Chemistry, Faculty of Chemistry, University of Murcia Regional Campus of International Excellence Campus Mare Nostrum, Campus of Espinardo, 30100 Murcia, Spain

[c] National Centre for Environmental Health (CNSA), Instituto de Salud Carlos III (ISCIII), Madrid, Spain.

[d] Department of Physical, Earth and Environmental Sciences, University of Siena, Via Mattioli, 4, 53100 Siena, Italy.

Correspondence: pam11@um.es

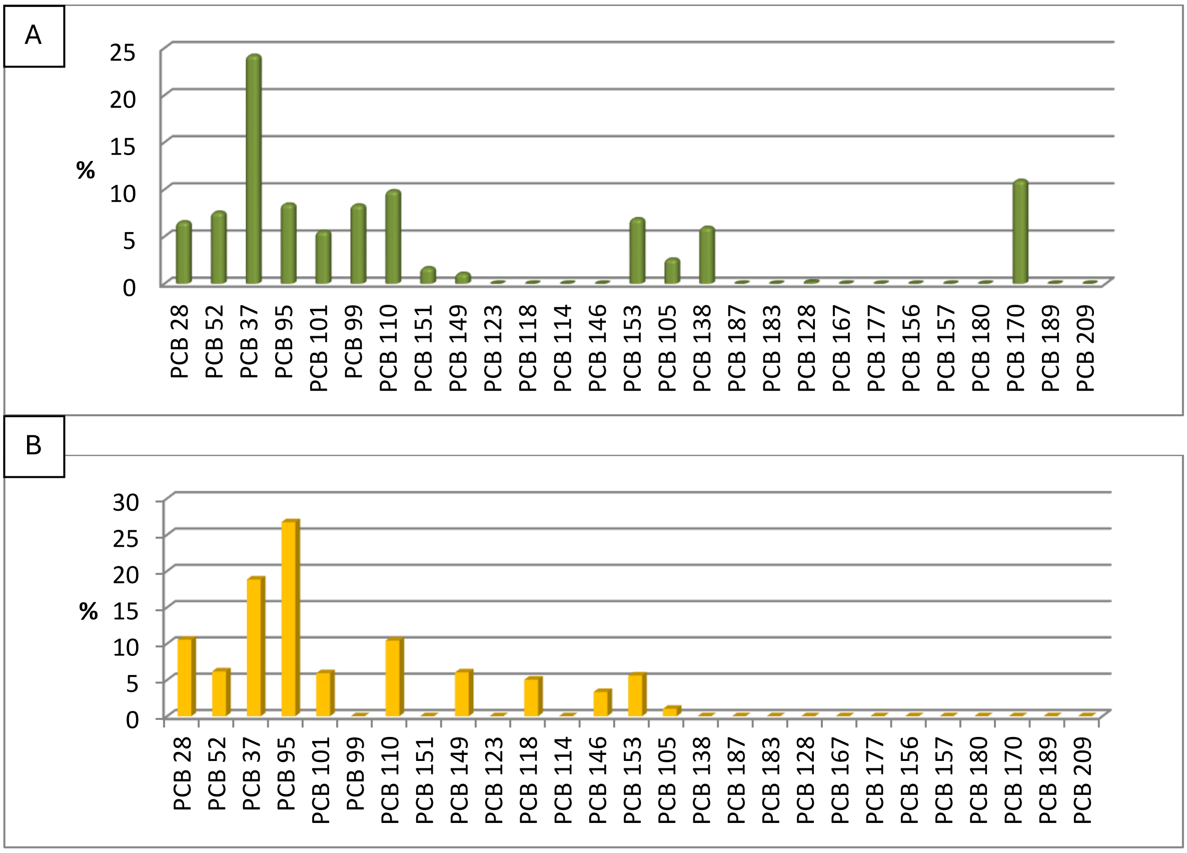

Figure S1. Fingerprints in liver of (A) adults and (B) chicks (results on w.w. basis).

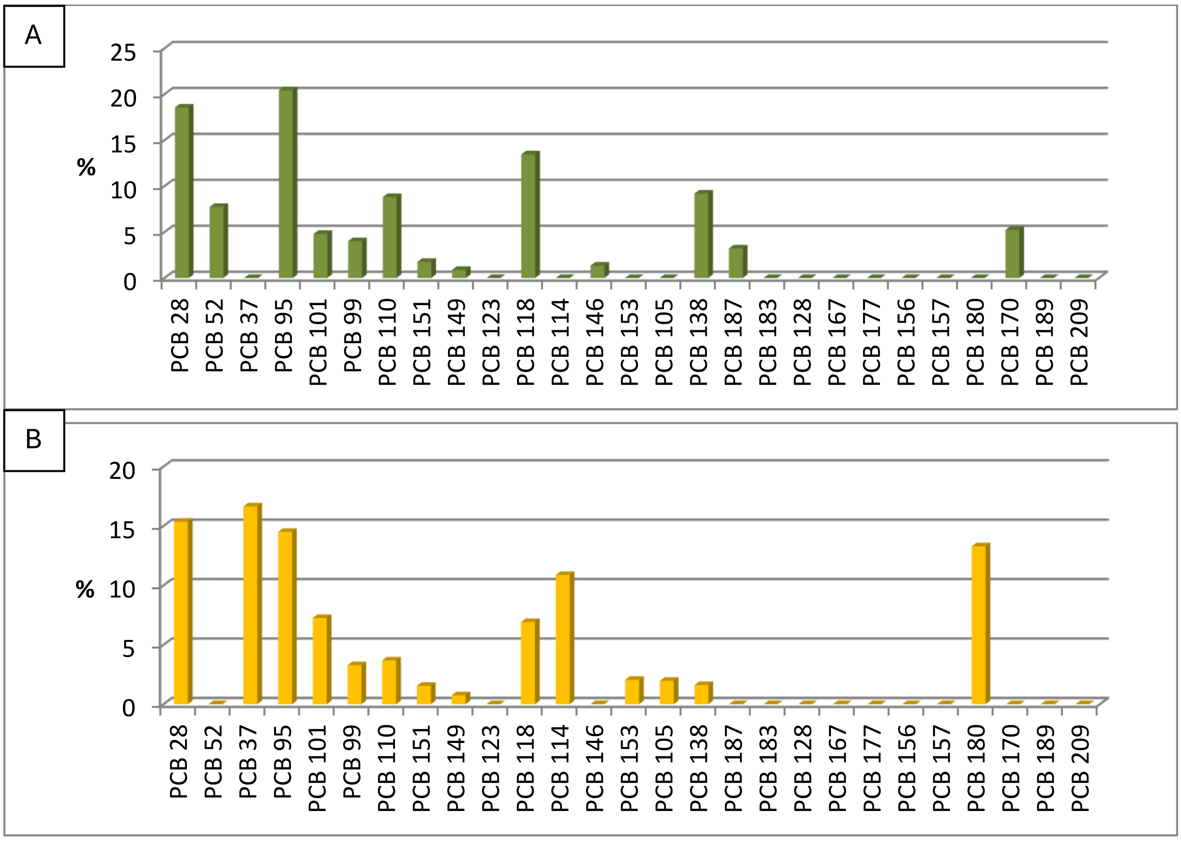

Figure S2. Fingerprints in kidney of (A) adults and (B) chicks (results on w.w. basis).

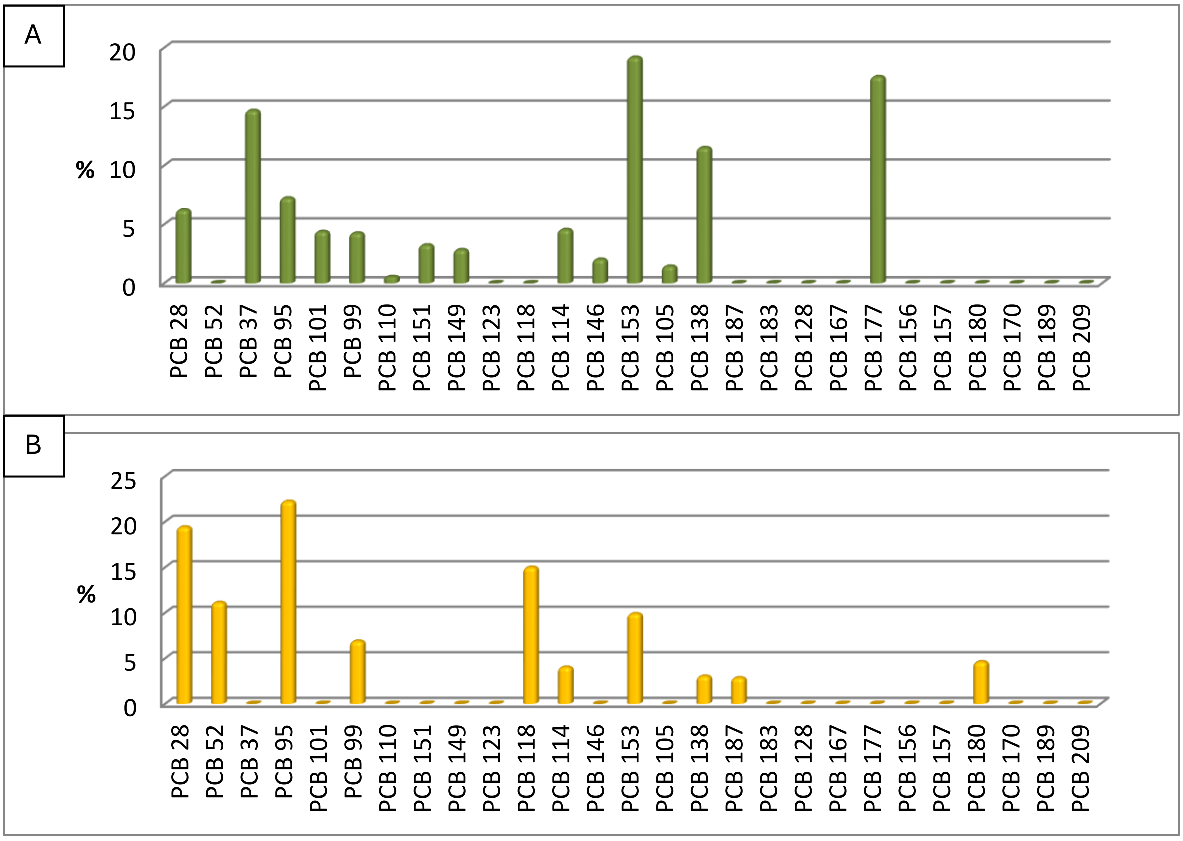

Figure S3. Fingerprints in muscle of (A) adults and (B) chicks (results on w.w. basis).

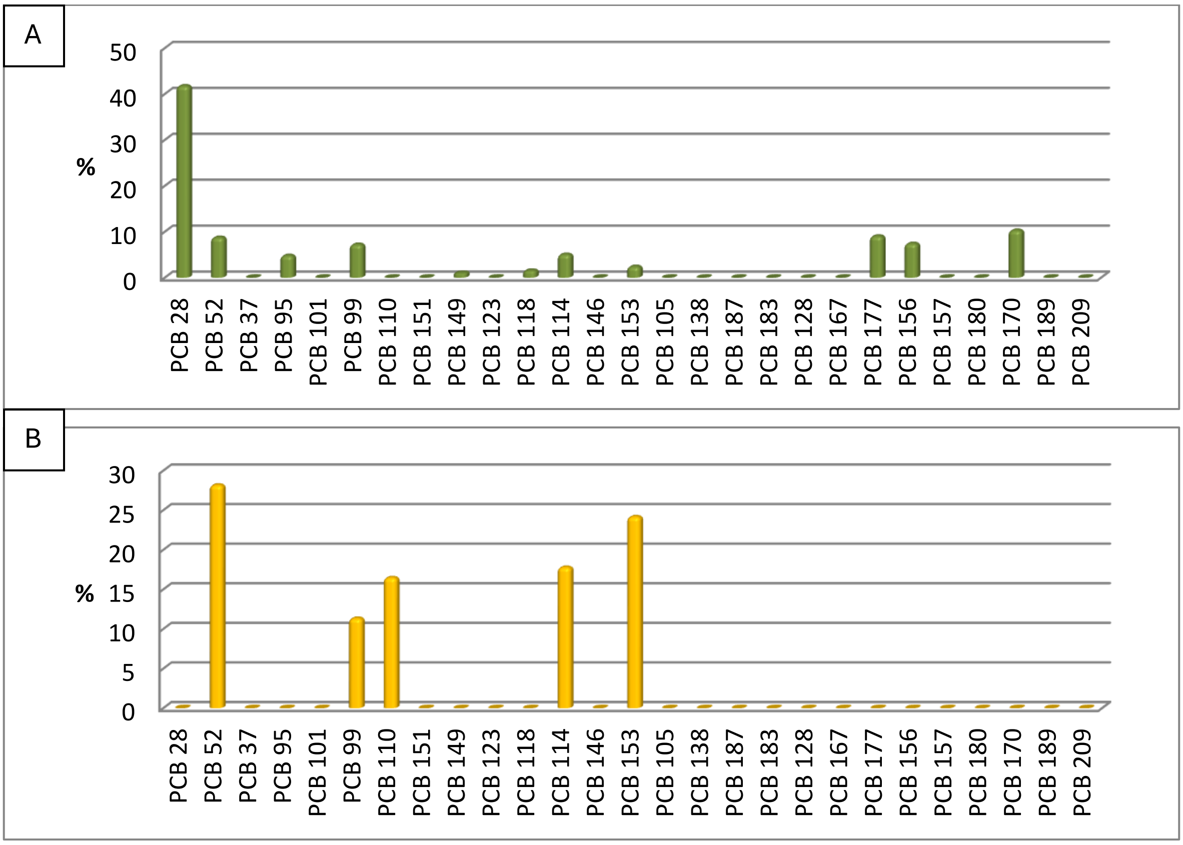

Figure S4. Fingerprints in heart of (A) adults and (B) chicks (results on w.w. basis).

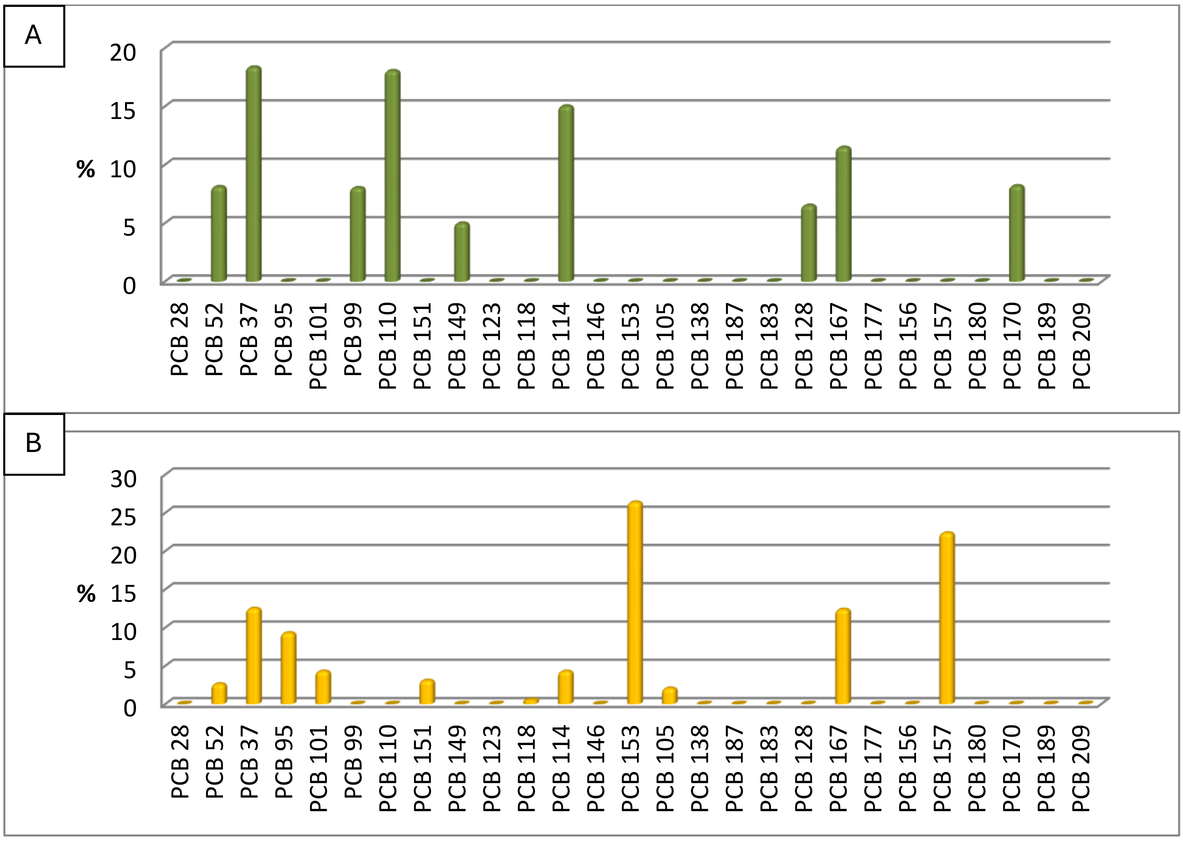

Figure S5. Fingerprints in brain of (A) adults and (B) chicks (results on w.w. basis).

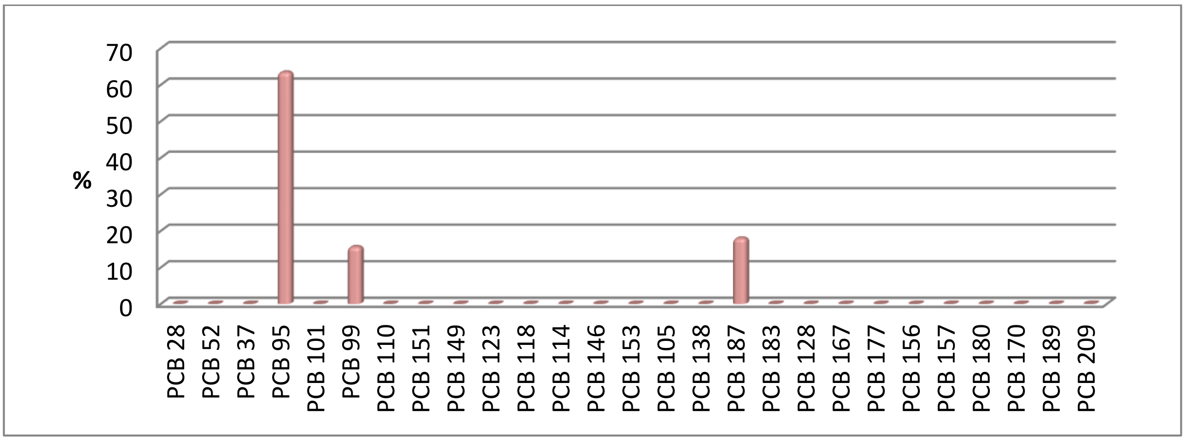

Figure S6. Fingerprints in krill (results on w.w. basis).

Table S1. PCB congeners studied in the works of Table 1.

PCB congeners	Reference
31, 44, 52, 77, 87, 101, 103, 105, 114, 118, 121, 128, 129, 137, 138, 141, 151, 153, 154, 156, 159, 170, 171, 180, 182, 183, PCB-185, 187, 189, 191, 195, 203, 205 and 207	[1]
138, 153 and 180	[2]
5, 8, 18, 31, 28, 33, 52, 49, 44, 74, 70, 66, 95, 84, 101, 99, 110, 77, 149, 118, 153, 105, 163, 138, 126, 187, 183, 128, 180, 200 and 194	[3]
138 and 153	[4]
99, 105, 118, 128, 138, 153, 170, 180, 183 and 87	[5]
77, 81, 126, 160, 118, 156 and 189	[6]
28, 31, 74, 95, 99, 101, 105, 110, 118, 128, 138, 149, 153, 156, 163, 170, 180, 183, 187, 194, 196 and 199	[7]
52, 99, 153 and 183	[8]
77, 81, 126, and 16	[9]
28, 52, 95, 101, 123, 118, 114, 153, 105, 138, 156, 157, 180 and 189	[10]
52, 101, 99, 149, 118, 153, 105, 138, 187, 183, 128, 156, 157, 180, 170,194 and 206	[11]
8, 52, 101, 118 + 149, 138, 153, and 180	[12]
8, 18, 28, 52, 44, 66, 101, 114, 105, 123,118, 153, 138, 187, 128, 167, 156, 157, 180, 170, 189, 195 and 206	[13]
8, 18, 26, 28, 44, 49, 50, 52, 66, 87, 101, 105, 110, 118, 128, 138/160, 149, 151, 153, 157, 173, 180, 183, 187, 194 and 195	[14]
8/5, 18, 29, 28/31, 52, 44, 66/95, 101/90, 87/115, 118, 153/132, 105, 138/160, 187, 128, 180, 170/190, 195/208, 206 and 209	[15]
8, 44, 52, 101, 118, 128, 138, 153, 180 and 187	[16]
8, 18, 28, 31, 33, 44, 49, 52, 56, 60, 66, 70, 74, 77, 87, 95, 97, 99, 101, 105, 110, 114, 118, 123, 126, 128, 132, 138, 141, 149, 151, 153, 156, 157, 158, 167, 169, 170, 174, 177, 180, 183, 187, 189, 194, 195, 199, 201, 203, 206 and 209	[17]
138, 153, 170, 180, 183 and 187	[18]
8, 18, 31, 28, 33, 52, 49, 44, 74, 70, 66, 95, 56/60, 101, 99, 97, 81, 87, 77, 110, 151, 123, 149, 118, 114, 153, 132, 105, 141, 138, 158, 126, 187, 183, 128, 167, 174, 177, 156, 157, 180, 169, 170, 199, 203, 189, 195, 194, 206 and 209	[19]
18, 52, 44, 101, 118, 153, 138, 187, 128 and 180	[20]

118, 138, 153 and 180	[21]
-	[22]
151, 149, 118, 146, 153, 141,138, 128, 174, 181,183, 177, 156, 171,202, 180, 199, 201,195, 170, 196, 194, and 206	[23]
81, 77, 126, 169, 105, 114, 118, 123, 156,157, 167, 189, 170 and 180	[24]
31/28, 21/20/33, 22, 52, 49, 48/47/75, 74, 70, 66/80, 101, 99, 123, 118, 105, 139/149, 146, 153/168, 164/163, 138, 156, 187/182, 183, 177, 172, 180/193 and 189	[25]
28, 20/33, 22, 52, 49, 48, 74, 70, 66, 101, 99, 123,118,114, 105, 149, 146, 153, 164, 138, 167, 156, 157, 178, 187, 183, 177, 172, 180, 170 and 189	[26]
118, 138, 153 and 180	[27]
28, 52, 77, 81, 101, 105, 114, 118, 123, 126, 138, 153, 156, 157, 167, 169, 18 and 189	[28]
28, 52, 77, 101, 118, 126, 138, 153, 169, and 180	[29]
28, 52, 101, 105, 118, 138, 153, 156, 180 and 209	[30]
77, 81, 105, 114, 118, 123, 126, 156, 157, 167, 169 and 189	[31]
8,18,28, 31, 33, 44, 49, 52, 56, 60, 66, 70, 74, 77, 81, 87, 95, 97, 99, 101, 105, 110, 114, 118, 123, 126, 128, 132, 138, 141, 149, 151, 153, 156, 157, 158, 167, 169, 170, 174, 177, 180, 183, 187, 189, 194, 195, 201, 203, 206 and 209	[32]
101, 118, 138, 153, 180 and 194	[33]
28, 52, 101, 118, 138, 153 and 180	[34]
4, 7, 20, 24, 28, 33, 34, 40, 42, 44, 52, 60, 70, 83, 85, 99, 101, 102, 105, 110, 118, 128, 133, 134, 137, 138, 141, 151, 153, 156, 70, 172, 174, 176, 177, 178, 180, 183, 187, 194,195,196, 201, 206 and 207	[35]
28, 31, 44, 45, 52, 66, 77/110, 87, 97, 101/90, 107/108, 118, 128, 132/105, 138/163/164, 141, 146, 149, 151, 153, 170, 174, 177, 179, 180, 183, 187/182, 194, 196 and 206	[36]
28, 52, 101, 118, 138, 153 and 180	[37]
8/5, 18, 28, 52, 44, 66/95, 90/101, 110/77, 149/106, 118, 132/153, 105, 138/160, 187, 128, 180, 170/190 and 206	[38]
28, 52, 101, 105, 118, 138, 153, 156, 180 and 209	[39]
18, 28, 31, 44, 52, 99, 101, 146, 149, 118, 153, 138, 163, 180, 170 and 194	[40]
Aroclor 1254 and Aroclor 1260	[41]

References

1. Rudolph, I.; Chiang, G.; Galban-Malagon, C.; Mendoza, R.; Martinez, M.; Gonzalez, C.; Becerra, J.; Servos, M.R.; Munkittrick, K.R.; Barra, R. Persistent organic pollutants and porphyrins biomarkers in penguin. faeces from Kopaitic Island and Antarctic Peninsula. *Scienceof the Total Environment* **2016**, *573*, 1390-1396, doi:https://doi.org/10.1016/j.scitotenv.2016.07.091.
2. Jara-Carrasco, S.; Barra, R.; Espejo, W.; Celis, J.E.; Gonzalez-Acuna, D.; Chiang, G.; Sanchez-Hernandez, J. Persistent organic pollutants and porphyrin levels in excreta of penguin colonies from the Antarctic Peninsula area. *Polar Record* **2017**, *53*, 79-87, doi:10.1017/s0032247416000607.
3. Adkesson, M.J.; Levengood, J.M.; Scott, J.W.; Schaeffer, D.J.; Langan, J.N.; Cárdenas-Alayza, S.; de la Puente, S.; Majluf, P.; Yi, S. Assessment of polychlorinated biphenyls, organochlorine pesticides, and polybrominated diphenyl ethers in the blood of humboldt penguins (Spheniscus humboldti) from the Punta San Juan marine protected area, Peru. *Journal of Wildlife Diseases* **2018**, *54*, 304-314, doi:https://doi.org/10.7589/2016-12-270.
4. Adkesson, M.J.; Shlosberg, A.; Lehner, A.F.; Rumbeiha, W.K.; Cardenas-Alayza, S.; Cardena-Mormontoy, M.; Kannan, K. Measurement of persistent organic pollutants, perfluorinated compounds, and toxic metals in the blood of humboldt penguins (Spheniscus humboldti) at Punta San Juan, Peru using dried blood spots. *Journal of Zoo and Wildlife Medicine* **2023**, *54*, 713-720, doi:https://doi.org/10.1638/2023-0047.
5. Dehnhard, N.; Jaspers, V.L.B.; Demongin, L.; Van den Steen, E.; Covaci, A.; Pinxten, R.; Crossin, G.T.; Quillfeldt, P.; Eens, M.; Poisbleau, M. Organohalogenated contaminants in plasma and eggs of rockhopper penguins: Does vitellogenin affect maternal transfer? *ENVIRONMENTAL POLLUTION* **2017**, *226*, 277-287, doi:https://doi.org/10.1016/j.envpol.2017.03.071.
6. Corsolini, S.; Borghesi, N.; Schiamone, A.; Focardi, S. Polybrominated diphenyl ethers, polychlorinated dibenzo-dioxins, -furans, and -biphenyls in three species of antarctic penguins. *Environmental Science and Pollution Research* **2007**, *14*, 421-429, doi:https://doi.org/10.1065/espr2006.01.017.
7. Van den Steen, E.; Poisbleau, M.; Demongin, L.; Covaci, A.; Dirtu, A.C.; Pinxten, R.; van Noordwijk, H.J.; Quillfeldt, P.; Eens, M. Organohalogenated contaminants in eggs of rockhopper penguins (Eudyptes chrysocome) and imperial shags (Phalacrocorax atriceps) from the Falkland Islands. *Sci. Total Environ.* **2011**, *409*, 2838-2844, doi:https://doi.org/10.1016/j.scitotenv.2011.04.002.
8. Corsolini, S.; Covaci, A.; Ademollo, N.; Focardi, S.; Schepens, P. Occurrence of organochlorine pesticides (OCPs) and their enantiomeric signatures, and concentrations of polybrominated diphenyl ethers (PBDEs) in the Adélie penguin food web, Antarctica. *Environmental Pollution* **2006**, *140*, 371-382, doi:https://doi.org/10.1016/j.envpol.2005.04.039.
9. Corsolini, S.; Kannan, K.; Imagawa, T.; Focardi, S.; Giesy, J.P. Polychloronaphthalenes and Other Dioxin-like Compounds in Arctic and Antarctic Marine Food Webs. *Environmental Science & Technology* **2002**, *36*, 3490-3496, doi:https://doi.org/10.1021/es025511v.
10. Mello, F.V.; Roscales, J.L.; Guida, Y.S.; Menezes, J.F.S.; Vicente, A.; Costa, E.S.; Jimenez, B.; Torres, J.P.M. Relationship between legacy and emerging organic pollutants in Antarctic seabirds and their foraging ecology as shown by δ13C and δ15N. *Sci. Total Environ.* **2016**, *573*, 1380-1389, doi:https://doi.org/10.1016/j.scitotenv.2016.07.080.
11. Bouwman, H.; Govender, D.; Underhill, L.; Polder, A. Chlorinated, brominated and fluorinated organic pollutants in African Penguin eggs: 30 years since the previous

assessment. *Chemosphere* **2015**, *126*, 1-10, doi:https://doi.org/10.1016/j.chemosphere.2014.12.071.
12. Corsolini, S.; Borghesi, N.; Ademollo, N.; Focardi, S. Chlorinated biphenyls and pesticides in migrating and resident seabirds from East and West Antarctica. *Environment International* **2011**, *37*, 1329-1335, doi:https://doi.org/10.1016/j.envint.2011.05.017.
13. Schiavone, A.; Corsolini, S.; Borghesi, N.; Focardi, S. Contamination profiles of selected PCB congeners, chlorinated pesticides, PCDD/Fs in Antarctic fur seal pups and penguin eggs. *Chemosphere* **2009**, *76*, 264-269, doi:https://doi.org/10.1016/j.chemosphere.2009.03.007.
14. Cipro, C.V.Z.; Taniguchi, S.; Montone, R.C. Occurrence of organochlorine compounds in Euphausia superba and unhatched eggs of Pygoscelis genus penguins from Admiralty Bay (King George Island, Antarctica) and estimation of biomagnification factors. *Chemosphere* **2010**, *78*, 767-771, doi:https://doi.org/10.1016/j.chemosphere.2009.10.006.
15. Quinete, N.; Hauser-Davis, R.A.; Lemos, L.S.; Moura, J.F.; Siciliano, S.; Gardinali, P.R. Occurrence and tissue distribution of organochlorinated compounds and polycyclic aromatic hydrocarbons in Magellanic penguins (Spheniscus magellanicus) from the southeastern coast of Brazil. *Sci. Total Environ.* **2020**, *749*, 141473, doi:https://doi.org/10.1016/j.scitotenv.2020.141473.
16. Baldassin, P.; Taniguchi, S.; Gallo, H.; Silva, R.J.; Montone, R.C. Persistent organic pollutants in juvenile Magellan penguins (Spheniscus magellanicus) found on the northern shore of the state of Sao Paulo and southern shore of the state of Rio de Janeiro, Brazil. *Marine Pollution Bulletin* **2012**, *64*, 2502-2506, doi:https://doi.org/10.1016/j.marpolbul.2012.07.035.
17. Baldassin, P.; Taniguchi, S.; Gallo, H.; Maranho, A.; Kolesnikovas, C.; Amorim, D.B.; Mansilla, M.; Navarro, R.M.; Tabeira, L.C.; Bicego, M.C.; et al. Persistent organic pollutants in juvenile Magellanic Penguins (Spheniscus magellanicus) in South America. *Chemosphere* **2016**, *149*, 391-399, doi:https://doi.org/10.1016/j.chemosphere.2016.01.016.
18. Taniguchi, S.; Montone, R.C.; Bicego, M.C.; Colabuono, F.I.; Weber, R.R.; Sericano, J.L. Chlorinated pesticides, polychlorinated biphenyls and polycyclic aromatic hydrocarbons in the fat tissue of seabirds from King George Island, Antarctica. *MARINE POLLUTION BULLETIN* **2009**, *58*, 129-133, doi:10.1016/j.marpolbul.2008.09.026.
19. Montone, R.C.; Taniguchi, S.; Colabuono, F.I.; Martins, C.C.; Cipro, C.V.Z.; Barroso, H.S.; da Silva, J.; Bícego, M.C.; Weber, R.R. Persistent organic pollutants and polycyclic aromatic hydrocarbons in penguins of the genus Pygoscelis in Admiralty Bay — An Antarctic specially managed area. *Marine Pollution Bulletin* **2016**, *106*, 377-382, doi:https://doi.org/10.1016/j.marpolbul.2016.02.047.
20. Inomata, O.N.K.; Montone, R.C.; Lara, W.H.; Weber, R.R.; Toledo, H.H.B. Tissue distribution of organochlorine residues - PCBs and pesticides - In Antarctic penguins. *Antarctic Science* **1996**, *8*, 253-255, doi:10.1017/s0954102096000351.
21. Jara-Carrasco, S.; Gonzalez, M.; Gonzalez-Acuna, D.; Chiang, G.; Celis, J.; Espejo, W.; Mattatall, P.; Barra, R. Potential immunohaematological effects of persistent organic pollutants on chinstrap penguin. *Antarctic Science* **2015**, *27*, 373-381, doi:10.1017/s0954102015000012.
22. Dekock, A.C.; Randall, R.M. ORGANOCHLORINE INSECTICIDE AND POLYCHLORINATED BIPHENYL RESIDUES IN EGGS OF COASTAL BIRDS FROM THE EASTERN CAPE, SOUTH-AFRICA. *Environmental Pollution Series a-Ecological and Biological* **1984**, *35*, 193-201, doi:10.1016/0143-1471(84)90202-2.

23. Court, G.S.; Davies, L.S.; Focardi, S.; Bargargli, R.; Fossi, C.; Leonzio, C.; Marili, L. Chlorinated hydrocarbons in the tissues of South Polar Skuas (Catharacta maccormicki) and Adelie Penguins (Pygoscelis adeliea) from Ross Sea, Antarctica. *Environmental Pollution* **1997**, *97*, 295-301, doi:10.1016/s0269-7491(97)00080-8.
24. Kumar, K.S.; Kannan, K.; Corsolini, S.; Evans, T.; Giesy, J.P.; Nakanishi, J.; Masunaga, S. Polychlorinated dibenzo-p-dioxins, dibenzofurans and polychlorinated biphenyls in polar bear, penguin and south polar skua. *Environmental Pollution* **2002**, *119*, 151-161, doi:https://doi.org/10.1016/S0269-7491(01)00332-3.
25. Kim, J.-T.; Choi, Y.-J.; Barghi, M.; Kim, J.-H.; Jung, J.-W.; Kim, K.; Kang, J.-H.; Lammel, G.; Chang, Y.-S. Occurrence, distribution, and bioaccumulation of new and legacy persistent organic pollutants in an ecosystem on King George Island, maritime Antarctica. *J. Hazard. Mater.* **2021**, *405*, 124141, doi:https://doi.org/10.1016/j.jhazmat.2020.124141.
26. Kim, J.-T.; Son, M.-H.; Kang, J.-H.; Kim, J.-H.; Jung, J.-W.; Chang, Y.-S. Occurrence of Legacy and New Persistent Organic Pollutants in Avian Tissues from King George Island, Antarctica. *Environmental Science & Technology* **2015**, *49*, 13628-13638, doi:https://doi.org/10.1021/acs.est.5b03181.
27. Jara, S.; Celis, J.E.; Araneda, A.; Gonzalez, M.; Espejo, W.; Barra, R. Assessment of persistent organic pollutants and their relationship with immunoglobulins in blood of penguin colonies from Antarctica. *Austral Journal of Veterinary Sciences* **2018**, *50*, 43-49, doi:10.4067/s0719-81322018000100108.
28. Morales, P.; Roscales, J.L.; Muñoz-Arnanz, J.; Barbosa, A.; Jiménez, B. Evaluation of PCDD/Fs, PCBs and PBDEs in two penguin species from Antarctica. *Chemosphere* **2022**, *286*, 131871, doi:https://doi.org/10.1016/j.chemosphere.2021.131871.
29. Souza, J.S.; Pacyna-Kuchta, A.D.; Teixeira da Cunha, L.S.; Costa, E.S.; Niedzielski, P.; Machado Torres, J.P. Interspecific and intraspecific variation in organochlorine pesticides and polychlorinated biphenyls using non-destructive samples from Pygoscelis penguins. *Environmental Pollution* **2021**, *275*, 116590, doi:https://doi.org/10.1016/j.envpol.2021.116590.
30. Lewis, P.J.; McGrath, T.J.; Chiaradia, A.; McMahon, C.R.; Emmerson, L.; Allinson, G.; Shimeta, J. A baseline for POPs contamination in Australian seabirds: little penguins vs. short-tailed shearwaters. *Marine Pollution Bulletin* **2020**, *159*, doi:10.1016/j.marpolbul.2020.111488.
31. Mwangi, J.K.; Lee, W.-J.; Wang, L.-C.; Sung, P.-J.; Fang, L.-S.; Lee, Y.-Y.; Chang-Chien, G.-P. Persistent organic pollutants in the Antarctic coastal environment and their bioaccumulation in penguins. *Environmental Pollution* **2016**, *216*, 924-934, doi:https://doi.org/10.1016/j.envpol.2016.07.001.
32. Colabuono, F.I.; Taniguchi, S.; Petry, M.V.; Montone, R.C. Organochlorine contaminants and polybrominated diphenyl ethers in eggs and embryos of Antarctic birds. *Antarctic Science* **2015**, *27*, 355-361, doi:10.1017/s0954102014000807.
33. Zhang, H.; Wang, Z.; Lu, B.; Zhu, C.; Wu, G.; Vetter, W. Occurrence of organochlorine pollutants in the eggs and dropping-amended soil of Antarctic large animals and its ecological significance. *Science in China Series D-Earth Sciences* **2007**, *50*, 1086-1096, doi:10.1007/s11430-007-0021-0.
34. Pala, N.; Vorkamp, K.; Bossi, R.; Ancora, S.; Ademollo, N.; Baroni, D.; Sara, G.; Corsolini, S. Chemical threats for the sentinel Pygoscelis adeliae from the Ross Sea (Antarctica): Occurrence and levels of persistent organic pollutants (POPs), perfluoroalkyl substances (PFAS) and mercury within the largest marine protected area worldwide. *Science of the Total Environment* **2024**, *947*, doi:10.1016/j.scitotenv.2024.174562.

35. Focardi, S.; Bargagli, R.; Corsolini, S. Isomer-specific analysis and toxic potential evaluation of polychlorinated biphenyls in Antarctic fish, seabirds and Weddell seals from Terra Nova Bay (Ross Sea). *Antarctic Science* **1995**, *7*, 31-35, doi:https://doi.org/10.1017/S095410209500006X.
36. van den Brink, N.W.; van Franeker, J.A.; de Ruiter-Dijkman, E.M. Fluctuating concentrations of organochlorine pollutants during a breeding season in two Antarctic seabirds: Adelie penguin and southern fulmar. *Environmental Toxicology and Chemistry* **1998**, *17*, 702-709, doi:https://doi.org/10.1002/etc.5620170426.
37. Pala, N.; Vorkamp, K.; Bossi, R.; Bignert, A.; Traversa, G.; Fugazza, D.; Ancora, S.; Ademollo, N.; Baroni, D.; Corsolini, S. Temporal trends of persistent organic pollutants (POPs) and perfluoroalkyl substances (PFAS) in Adelie penguin (Pygoscelis adeliae) eggs from the Ross Sea (Antarctica), including their relationship with climate parameters. *Environmental Pollution* **2025**, *373*, doi:https://doi.org/10.1016/j.envpol.2025.126130.
38. Terajima, T.; Shibahara, A.; Nakano, Y.; Kobayashi, S.; Godwin, J.R.; Nagaoka, K.; Watanabe, G.; Takada, H.; Mizukawa, K. Age-related accumulation of persistent organic chemicals in captive king penguins (<i>Aptenodytes patagonicus</i>). *Journal of Veterinary Medical Science* **2022**, *84*, 1551-1555, doi:10.1292/jvms.22-0245.
39. Lewis, P.J.; Lashko, A.; Chiaradia, A.; Allinson, G.; Shimeta, J.; Emmerson, L. New and legacy persistent organic pollutants (POPs) in breeding seabirds from the East Antarctic. *Environmental Pollution* **2022**, *309*, 119734, doi:https://doi.org/10.1016/j.envpol.2022.119734.
40. Weichbrodt, M.; Vetter, W.; Scholza, E.; Luckas, B.; Reinhardt, K. Determination of Organochlorine Levels in Antarctic Skua and Penguin Eggs by Application of Combined Focused Open-Vessel Microwave-Assisted Extraction, Gel-Permeation Chromatography, Adsorption Chromatography, and GC/ECD. *International Journal of Environmental Analytical Chemistry* **1999**, *73*, 309-328, doi:https://doi.org/10.1080/03067319908032672.
41. Luke, B.G.; Johnstone, G.W.; Woehler, E.J. ORGANOCHLORINE PESTICIDES, PCBS AND MERCURY IN ANTARCTIC AND SUB-ANTARCTIC SEABIRDS. *Chemosphere* **1989**, *19*, 2007-2021, doi:10.1016/0045-6535(89)90024-6.
